# Hip Fracture Patterns Among Hispanic Seniors: Risk Factors and Implications

**DOI:** 10.7759/cureus.80463

**Published:** 2025-03-12

**Authors:** Nikhil Mathur, John Knight, Monica Betancourt-Garcia, Gregery Pequeno, Michael Serra-Torres

**Affiliations:** 1 Trauma Research, Doctors Hospital at Renaissance Health System, Institute for Research and Development, Edinburg, USA; 2 Orthopedics and Trauma, Doctors Hospital at Renaissance Health System, Edinburg, USA

**Keywords:** extracapsular fracture, geriatric, hip fracture, hispanic, intracapsular fracture

## Abstract

Background

Hip fractures are a major cause of morbidity and mortality in the growing US geriatric population, with the majority resulting from falls. They are associated with a significant loss of independence and impose a substantial financial burden on healthcare systems worldwide. The Rio Grande Valley (RGV), a medically underserved region with a predominantly Hispanic population, faces high rates of chronic conditions such as diabetes and obesity, which may influence fracture patterns and outcomes. This study examines hip fractures in a predominantly Hispanic geriatric cohort, focusing on the impact of diabetes and obesity on fracture type, with the goal of informing targeted prevention and treatment strategies.

Methods

This retrospective cohort study was conducted at a Level 1 Trauma Center along the US-Mexico border. The study included hip fracture cases caused by falls in patients aged 65 and older over a three-year period, excluding periprosthetic and pathologic fractures. Fractures were stratified as intracapsular (femoral head/neck) or extracapsular (intertrochanteric, subtrochanteric, and greater/lesser trochanter). Treatment strategies included arthroplasty, osteosynthesis, or conservative management. Outcome measures included one-year all-cause mortality, length of stay (LOS), readmission rates, and complications such as deep vein thrombosis (DVT), pulmonary embolism (PE), fat embolism, pressure ulcers, and surgical site infections (SSIs). Statistical analyses assessed associations between fracture type, patient characteristics, treatment strategies, and outcomes.

Results

The study included 412 patients, of whom 85.2% (351) were Hispanic and 71.4% (294) were female, with a mean age of 80.6 years and a body mass index (BMI) of 25.5 kg/m^2^. Higher age (mean: 81.3 years, p=0.033), lower BMI (25.0 vs. 26.2, p=0.019), and Hispanic ethnicity (OR: 1.98, p=0.026) were associated with extracapsular fractures. Non-surgical management was associated with a significantly higher one-year mortality rate (n=6; 20.7%; p=0.004). Surgery performed more than 48 hours after arrival prolonged hospital stay (7.96 vs. 5.73 days for <24 hours, p<0.001). The overall one-year mortality rate was 5.6% (23), with older age (OR: 1.08, p=0.034), COPD (OR: 5.24, p=0.015), and cirrhosis (OR: 8.69, p=0.024) as significant predictors. Prolonged immobilization (OR: 2.68, p=0.016) and diabetes (OR: 3.89, p=0.002) increased complication rates.

Conclusion

Aging, comorbidities, and Hispanic ethnicity increased extracapsular fracture risk, while a higher BMI was predictive for intracapsular fractures. The one-year mortality rate of 5.6% highlighted the Hispanic paradox, suggesting a survival advantage despite the presence of multiple comorbidities and risk factors. Ultimately, these findings emphasize the necessity of targeted intervention strategies, including fall prevention programs, bone health education, and culturally tailored healthcare approaches. Addressing ethnic and socioeconomic disparities in osteoporosis screening and fracture management remains essential for improving outcomes and reducing hip fracture occurrence within this high-risk population.

## Introduction

According to the 2020 US Census, geriatric individuals (≥65 years of age) accounted for 16.81% (55.8 million) of the population [1]. As life expectancy increases, this demographic is projected to exceed 94 million by 2060 [2]. Geriatric patients face a heightened risk of hip fractures, which are linked to high morbidity and mortality [3-10]. Annually, about 300,000 hip fractures occur among this group, with falls causing 90% of these injuries [3-5,11-16]. Hip fractures lead to reduced mobility, loss of independence, and a decline in quality of life. Additionally, underlying medical conditions significantly contribute to post-fracture mortality, with one-year mortality rates estimated at 20%-30% [5,7,10,14,16-20].

Hip fractures are divided into two types with distinct clinical implications: intracapsular (femoral head and neck) and extracapsular (intertrochanteric, subtrochanteric, and greater/lesser trochanter) fractures [12-14,17]. Fracture type influences treatment approaches, recovery outcomes, and long-term health effects [13,14,17,20]. Without proper management, these fractures can result in decreased mobility, loss of independence, and a significant decline in quality of life.

Risks for hip fractures are multifactorial, with primary contributors including age, female sex, osteoporosis, and falls [8,9,21-28]. In the US, hip fractures impose an annual economic burden of nearly $29 billion, covering hospitalization, surgery, rehabilitation, and long-term care [3,4,6,29]. The cost of hip fractures continues to rise with longer life expectancy and higher medical expenses, further exacerbated by ongoing care and rehabilitation costs [3-5,16,17,20,29].

Residents of the Rio Grande Valley (RGV), a border region in the most southern part of Texas, face unique health challenges shaped by socioeconomic factors, limited healthcare access, cultural influences, and proximity to the US-Mexico border. The predominantly Hispanic population (91%) includes a geriatric segment of 13%, many living in economically disadvantaged and medically underserved areas [30]. The region faces high rates of chronic conditions like osteoporosis, diabetes, obesity, hypertension, and cardiovascular disease, increasing the risk of complications such as hip fractures.

Geriatric hip fractures are often caused by ground-level falls, with intertrochanteric fractures being the most common [31-33]. Factors such as elevated BMI, microvascular disease (including diabetic neuropathy and microangiopathy), and diabetes-related hypoglycemia increase the risk of falls by affecting movement dynamics and compromising bone strength [6,8,9,34,35]. This study analyzes hip fractures from falls in a predominantly Hispanic geriatric population at a Level 1 Trauma Center along the US-Mexico border. Higher rates of diabetes and obesity in Hispanic geriatric patients are expected to increase the occurrence of intertrochanteric fractures [8,9,20,31-33]. The findings aim to enhance understanding of regional and demographic disparities in hip fracture risks and outcomes, informing targeted prevention and treatment strategies.

## Materials and methods

Data source and study population

A query was performed to identify hip fracture cases due to falls from September 1, 2021, to September 1, 2024, in patients aged ≥65 treated at a Level 1 Trauma Center along the US-Mexico border. The hospital trauma registry, which is used to collect, maintain, and report trauma data, along with Electronic Medical Records (EMR), were reviewed to extract patient information, while radiographic imaging was used to verify fracture type and management.

Study variables

Study variables were categorized into patient demographics, risk factors, outcomes, hip fracture diagnoses, and management strategies. Demographic data included age, ethnicity, sex, and BMI. Risk factors included comorbidities (cardiovascular disease, osteoporosis, diabetes mellitus, dementia, obesity, chronic obstructive pulmonary disease (COPD), peripheral arterial disease, cerebral vascular accident, and cirrhosis), alcohol use, smoking status, and history of fragility fractures. Outcomes were assessed by one-year all-cause mortality, hospital length of stay (LOS), readmission rates, and complication rates, which included deep vein thrombosis (DVT), pulmonary embolism (PE), fat embolism, pressure ulcers, and surgical site infections (SSIs). Fracture diagnoses were identified using the International Classification of Disease, Tenth Revision (ICD-10) codes (S72.0, S72.1, S72.2) and relevant modifiers. Hip fractures were classified as intracapsular (femoral head (OTA/AO 31C), femoral neck (OTA/AO 31B)) and extracapsular (OTA/AO 31A), including intertrochanteric, subtrochanteric, and trochanteric fractures [12]. Treatment strategies included arthroplasty, osteosynthesis, or conservative management.

Statistical analyses

Baseline characteristics of patients with hip fractures were summarized by fracture type (intracapsular vs. extracapsular) and the presence of osteoporosis. Numerical variables were compared using Student’s t-test (for normal distributions) or the Mann-Whitney U test (for non-normal distributions). Categorical variables were analyzed using chi-squared or Fisher’s exact tests as appropriate. Patients were stratified into age (65-74, 75-84, and ≥85 years) and BMI (underweight: <18.5, normal: 18.5-24.9, overweight: 25-29.9, and obese: ≥30 kg/m^2^) groups. Age and BMI categories were selected based on previous literature to facilitate analyses [36,37]. Fracture type was first compared by BMI category and sex using a side-by-side column chart, followed by a comparison by age category and sex using chi-squared tests. The neck-to-intertrochanteric (N:IT) ratio was calculated for femoral neck and intertrochanteric fractures to enable comparison with previous studies [36,37]. A two-proportion z-test was used to compare age-sex stratified groups and then adjusted for multiple comparisons using the Bonferroni correction. Patient outcomes, including one-year mortality, LOS, and post-fracture complications, were analyzed by BMI, fracture type, management type, ethnicity, and time to surgery. Categorical outcomes were analyzed using chi-squared or Fisher’s exact tests, and LOS was compared using the Kruskal-Wallis test. Logistic regression models were built to predict extracapsular fracture type, one-year mortality, and post-fracture complications, with predictors including demographics, comorbidities, and risk factors. Multicollinearity was assessed using variance inflation factors (VIFs), and model fit was evaluated using the Hosmer-Lemeshow test. Interaction terms for age, sex, and BMI were tested for significance. A power analysis indicated that a sample size of 227 patients would provide sufficient power to detect a two-day difference in LOS. A significance level of 0.05 was used for all tests. Statistical analyses were performed using R (version 4.4.2) (R Foundation for Statistical Computing, Vienna, Austria).

## Results

Demographics and comorbidities

There were 412 patients included in the study, of which 351 (85.2%) identified as Hispanic, while 61 (14.8%) were non-Hispanic. The mean age of the patients was 80.6 years (SD: 8.1), with a mean BMI of 25.5 kg/m^2^ (SD: 5.4). Female patients comprised 71.4% (294) of the cohort, while 28.6% (118) were male. Patients with extracapsular fractures were significantly older, with a mean of 81.3 years, compared to those with intracapsular fractures (79.6 years) (p=0.033) (Table 1). Additionally, patients with extracapsular fractures had a significantly lower mean BMI (25.0 vs. 26.2; p=0.019). A pre-existing diagnosis of osteoporosis was present in 95 patients (23.1%) who were significantly older (82.4 vs. 80.0 years; p=0.012) and more likely to be female (n=78; 82.1%; p=0.012) compared to those without osteoporosis (Table 2).

**Table 1:** Baseline characteristics of patients with hip fractures ^a^ χ^2^ = 0.419, 1 DF. ^b ^χ^2^ = 3.670, 1 DF.  ^c^ χ^2^ = 2.300, 1 DF.  ^d^ χ^2^ = 0.485, 1 DF.  ^e^ χ^2^ < 0.001, 1 DF. DF: degree of freedom; BMI: body mass index

Variable	Overall	Intracapsular	Extracapsular	P-value
N	412	180 (43.7%)	232 (56.3%)	
Age				
mean (SD)	80.6 (8.1)	79.6 (7.9)	81.3 (8.2)	0.033
BMI				
mean (SD)	25.5 (5.4)	26.2 (5.6)	25.0 (5.1)	0.019
Time to surgery (hours)				
mean (SD)	22.5 (13.4)	22.7 (13.8)	22.4 (13.1)	0.779
Sex				
Male	118 (28.6%)	55 (30.6%)	63 (27.2%)	0.517^a^
Female	294 (71.4%)	125 (69.4%)	169 (72.8%)	
Ethnicity				
Non-Hispanic	61 (14.8%)	34 (18.9%)	27 (11.6%)	0.055^b^
Hispanic	351 (85.2%)	146 (81.1%)	205 (88.4%)	
Fracture laterality				
Left	192 (46.6%)	92 (51.1%)	100 (43.1%)	0.129^c^
Right	220 (53.4%)	88 (48.9%)	132 (56.9%)	
Displacement				
Nondisplaced	64 (15.5%)	31 (17.2%)	33 (14.2%)	0.486^d^
Displaced	348 (84.5%)	149 (82.8%)	199 (85.8%)	
Fall Height >1 m				
No	371 (96.4%)	158 (96.3%)	213 (96.4%)	1.000^e^
Yes	14 (3.6%)	6 (3.7%)	8 (3.6%)	

**Table 2:** Baseline characteristics of patients with and without osteoporosis ^a^ χ^2^ = 6.310, 1 DF. ^b^ χ^2^ = 0.643, 1 DF. ^c^ χ^2^ = 0.892, 1 DF. ​​​​​​​^d^ χ^2^ = 0.983, 1 DF. ​​​​​​​^e^ χ^2^ = 0.317, 1 DF. ​​​​​​​^f^ Fisher’s Exact test used. BMI: body mass index; DF: degree of freedom

Variable	Overall	No osteoporosis	Osteoporosis	P-value
N	412	317 (76.9%)	95 (23.1%)	
Age				
mean (SD)	80.6 (8.1)	80.0 (8.2)	82.4 (7.5)	0.012
BMI				
mean (SD)	25.5 (5.4)	25.5 (5.5)	25.6 (5.0)	0.817
Time to surgery (hours)				
mean (SD)	22.5 (13.4)	23.1 (14.6)	20.7 (7.6)	0.142
Sex				
Male	118 (28.6%)	101 (31.9%)	17 (17.9%)	0.012^a^
Female	294 (71.4%)	216 (68.1%)	78 (82.1%)	
Ethnicity				
Non-Hispanic	61 (14.8%)	44 (13.9%)	17 (17.9%)	0.423^b^
Hispanic	351 (85.2%)	273 (86.1%)	78 (82.1%)	
Fracture type				
Intracapsular	180 (43.7%)	143 (45.1%)	37 (38.9%)	0.345^c^
Extracapsular	232 (56.3%)	174 (54.9%)	58 (61.1%)	
Fracture laterality				
Left	192 (46.6%)	143 (45.1%)	49 (51.6%)	0.321^d^
Right	220 (53.4%)	174 (54.9%)	46 (48.4%)	
Displacement				
Nondisplaced	64 (15.5%)	47 (14.8%)	17 (17.9%)	0.574^e^
Displaced	348 (84.5%)	270 (85.2%)	78 (82.1%)	
Fall height >1 m				
No	371 (96.4%)	285 (96.0%)	86 (97.7%)	0.745^f^
Yes	14 (3.6%)	12 (4.0%)	2 (2.3%)	

**Figure 1:**
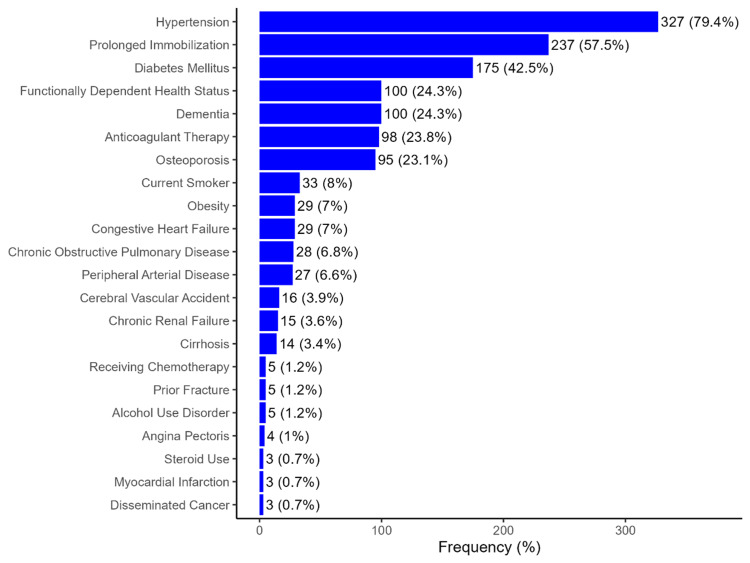
Comorbidities and risk factors in the patient cohort

Hypertension was the most common comorbidity in this cohort, affecting 327 patients (79.4%) (Figure 1). Other frequent comorbidities included prolonged immobilization (237; 57.5%), diabetes (175; 42.5%), functional dependency (100; 24.3%), dementia (100; 24.3%), anticoagulant therapy (98; 23.8%), and osteoporosis (95; 23.1%). Seven patients (1.7%) had no recorded comorbidities or risk factors.

Fracture type

Categorically, 232 patients (56.3%) had extracapsular fractures while 180 (43.7%) had intracapsular fractures. Intertrochanteric fractures were the most common (219; 53.2%), followed by femoral neck fractures (167; 40.5%), subtrochanteric fractures (17; 4.1%), greater trochanter fractures (10; 2.4%), and femoral head fractures (2; 0.5%). Lesser trochanter fractures were not observed in the cohort. There were three patients (0.73%) who experienced multiple fracture types.

Among Hispanic patients, 205 (58.4%) had extracapsular fractures and 146 (41.6%) had intracapsular fractures. Non-Hispanic patients exhibited the opposite trend, with 27 (44.3%) extracapsular and 34 (55.7%) intracapsular fractures. Extracapsular fractures were more common in patients aged ≥85 (n=86; 61.9%; p=0.268) among both males (n=25; 61%; p=0.408) and females (n=61; 62.2%; p=0.438); however, these differences were not statistically significant (Table 3).

**Table 3:** Distribution of fracture type by age and sex ^a^ χ^2^ = 2.636, 2 DF. ^b^ χ^2^ = 1.794, 2 DF. ^c^ χ^2^ = 1.650, 2 DF. DF: degree of freedom

Sex	Age	Intracapsular	Extracapsular	P-value
All	65-74	46 (46.5%)	53 (53.5%)	0.268^a^
	75-84	81 (46.6%)	93 (53.4%)	
	≥85	53 (38.1%)	86 (61.9%)	
Male	65-74	19 (54.3%)	16 (45.7%)	0.408^b^
	75-84	20 (47.6%)	22 (52.4%)	
	≥85	16 (39.0%)	25 (61.0%)	
Female	65-74	27 (42.2%)	37 (57.8%)	0.438^c^
	75-84	61 (46.2%)	71 (53.8%)	
	≥85	37 (37.8%)	61 (62.2%)	

Stratification by BMI revealed that intracapsular fractures were most frequent in patients with obesity (39; 52.7%), followed by overweight (65; 44.2%), normal weight (64; 40.5%), and underweight patients (12; 36.4%). 

**Figure 2:**
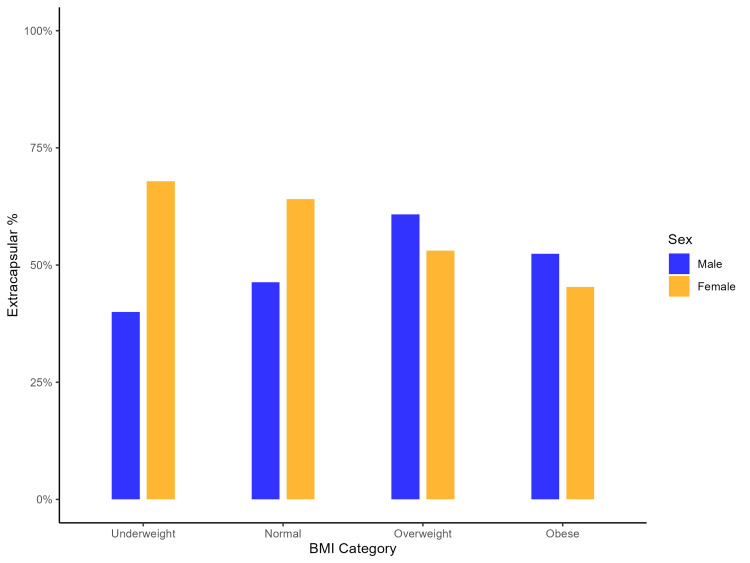
Distribution of extracapsular fractures by BMI category and sex BMI: body mass index

Logistic regression analysis (Table 4) demonstrated that Hispanic ethnicity (OR: 1.98; CI: 1.09-3.60; p=0.026) was associated with a higher likelihood of extracapsular fractures, while higher BMI (OR: 0.95; CI: 0.92-0.99; p=0.023) was associated with a decreased likelihood of extracapsular fractures. Additionally, a significant negative interaction was found between female sex and BMI (OR: 0.90, 95% CI: 0.82-0.98; p=0.016), as reflected in Figure 2.

**Table 4:** Logistic regression predicting extracapsular fracture type BMI: body mass index; COPD: chronic obstructive pulmonary disease

Variable	OR (95% CI)	P-value
Age	1.03 (1.00-1.06)	0.071
Sex=female	1.05 (0.66-1.69)	0.833
Ethnicity=Hispanic	1.98 (1.09-3.60)	0.026
BMI	0.95 (0.92-0.99)	0.023
Fall height >1 m	1.14 (0.36-3.58)	0.828
Hypertension	1.03 (0.60-1.77)	0.903
Prolonged immobilization	1.03 (0.68-1.56)	0.877
Diabetes mellitus	1.05 (0.67-1.63)	0.839
Functionally dependent health status	0.91 (0.55-1.53)	0.733
Dementia	0.70 (0.43-1.15)	0.163
Anticoagulant therapy	0.82 (0.50-1.35)	0.437
Osteoporosis	1.27 (0.78-2.09)	0.338
Current smoker	1.03 (0.48-2.23)	0.933
Obesity	0.98 (0.43-2.23)	0.956
Congestive heart failure	0.82 (0.35-1.91)	0.652
COPD	1.44 (0.60-3.44)	0.416
Peripheral arterial disease	0.67 (0.29-1.53)	0.342
Cerebral vascular accident	1.09 (0.38-3.10)	0.874
Chronic renal failure	0.90 (0.30-2.66)	0.848
Cirrhosis	1.38 (0.43-4.43)	0.585

Table 5 presents a comparative analysis of N:IT ratios between the study cohort and previous studies [36,37]. Among male patients in the study cohort, the N:IT ratio decreased with age, from 1.21 in the 65-74 age group to 0.90 in the 75-84 group, and 0.67 in those aged 85 and older. For female patients, the N:IT ratio was significantly lower in the 65-74 age group compared to the cohorts from Japan (0.79 vs. 2.31; p<0.001) and Sweden (0.79 vs. 1.79; p=0.002) [36,37]. However, no significant differences were observed for females in the 75-84 (0.98 N:IT) and ≥85 (0.62 N:IT) age groups compared to the other cohorts [36,37].

**Table 5:** Comparison of N:IT ratios across Edinburg, USA; Kyoto, Japan; and Östergötland, Sweden N:IT: neck-to-intertrochanteric

		Edinburg, USA	Kyoto, Japan	P-value	Östergötland, Sweden	P-value
Male	65-74	1.21 (17/14)	1.29 (17664/13680)	0.865	1.03 (256/249)	0.654
	75-84	0.90 (19/21)	0.99 (34042/34228)	0.765	1.26 (1007/799)	0.299
	≥85	0.67 (16/24)	0.79 (26496/33460)	0.594	1.29 (3156/2454)	0.039
Female	65-74	0.79 (26/33)	2.31 (46549/20123)	<0.001	1.79 (502/281)	0.002
	75-84	0.98 (59/60)	1.12 (116858/104372)	0.4788	1.22 (1848/1518)	0.252
	≥85	0.62 (36/58)	0.60 (124749/206694)	0.895	0.84 (4183/4954)	0.147

Management

There were 260 patients (63.1%) who underwent osteosynthesis, 123 (29.9%) who received arthroplasty, and 29 (7.0%) who were managed non-surgically (Table 6). The most common procedures were cephalomedullary nail fixation and hemiarthroplasty, each performed in 121 patients (29.4%). Other procedures included intramedullary nailing (107; 26.0%), percutaneous screw fixation (20; 4.9%), femoral neck system (11; 2.7%), total hip arthroplasty (2; 0.5%), and dynamic hip screw fixation (1; 0.2%).

**Table 6:** Patient outcomes ^a^ Fisher’s Exact test was used. ^b^ χ^2^ = 0.056, 1 DF. ^c^ χ^2^ = 0.220, 1 DF. ^d^ χ^2^ = 1.419, 1 DF. BMI: body mass index; DF: degree of freedom; LOS: length of stay

	n (%)	1 y mortality (rate)	P-value	Mean LOS (SD)	P-value	Complications (rate)	P-value
BMI Category			0.786^a^		0.731		0.055^a^
Underweight	33 (8.0)	3 (9.1)		5.76 (2.79)		8 (24.2)	
Normal	158 (38.3)	8 (5.1)		5.97 (3.00)		12 (7.6)	
Overweight	147 (35.7)	8 (5.4)		5.95 (2.90)		15 (10.2)	
Obese	74 (18.0)	4 (5.4)		6.17 (2.62)		6 (8.1)	
Fracture type			0.812^b^		0.293		0.639^c^
Intracapsular	180 (43.7)	9 (5.0)		5.85 (2.84)		16 (8.9)	
Extracapsular	232 (56.3)	14 (6.0)		6.09 (2.90)		25 (10.8)	
Procedure type			0.004^a^		0.670		0.641^a^
Osteosynthesis	260 (63.1)	10 (3.8)		6.03 (2.84)		24 (9.2)	
Arthroplasty	123 (29.9)	7 (5.7)		5.93 (2.69)		13 (10.6)	
Conservative management	29 (7.0)	6 (20.7)		5.77 (3.95)		4 (13.8)	
Ethnicity			0.761^a^		0.737		0.234^d^
Hispanic	351 (85.2)	19 (5.4)		5.95 (2.75)		38 (10.8)	
Non-Hispanic	61 (14.8)	4 (6.6)		6.20 (3.49)		3 (4.9)	
Time to surgery			0.315^a^		<0.001		0.444^a^
≤24 hours	269 (70.6)	10 (3.7)		5.73 (2.85)		25 (9.3)	
24-48 hours	97 (25.5)	7 (7.2)		6.44 (2.34)		11 (11.3)	
>48 hours	15 (3.9)	0 (0.0)		7.96 (3.29)		0 (0.0)	

Patient outcomes

A total of 41 patients (10.0%) experienced complications. All-cause mortality within one year of admission was reported in 23 patients (5.6%), while two patients (0.5%) were readmitted. The mean LOS was 6.1 days (SD: 3.4).

Prolonged immobilization (OR: 2.68; CI: 1.20-5.99; p=0.016), diabetes mellitus (OR: 3.89; CI: 1.67-9.07; p=0.002), and cirrhosis (OR: 6.87; CI: 1.66-28.41; p=0.008) were significantly associated with complications. Osteoporosis, however, was associated with a reduced risk of complications (OR: 0.32; CI: 0.10-0.99; p=0.048) (Table 7).

**Table 7:** Logistic regression predicting post-fracture complications BMI: body mass index; COPD: chronic obstructive pulmonary disease

Variable	OR (95% CI)	P-value
Age	1.01 (0.96-1.06)	0.705
Sex=female	0.83 (0.38-1.83)	0.645
Ethnicity=Hispanic	1.09 (0.29-4.18)	0.897
BMI	0.97 (0.90-1.04)	0.374
Displacement=displaced	4.06 (1.00-16.50)	0.050
Prior fracture	6.57 (0.55-78.03)	0.136
Time to procedure (hours)	0.98 (0.94-1.01)	0.219
Procedure type=arthroplasty	0.86 (0.38-1.94)	0.710
Procedure type=conservative management	0.78 (0.15-3.90)	0.760
Hypertension	1.33 (0.43-4.14)	0.625
Prolonged immobilization	2.68 (1.20-5.99)	0.016
Diabetes mellitus	3.89 (1.67-9.07)	0.002
Functionally dependent health status	1.01 (0.41-2.45)	0.985
Dementia	1.75 (0.74-4.15)	0.204
Anticoagulant therapy	2.03 (0.89-4.62)	0.091
Osteoporosis	0.32 (0.10-0.99)	0.048
Current smoker	0.81 (0.16-4.07)	0.802
Obesity	3.34 (0.95-11.72)	0.060
Congestive heart failure	1.86 (0.56-6.22)	0.313
COPD	0.75 (0.14-3.89)	0.731
Peripheral arterial disease	1.21 (0.34-4.30)	0.773
Cerebral vascular accident	1.12 (0.24-5.21)	0.881
Cirrhosis	6.87 (1.66-28.41)	0.008

Patients treated conservatively exhibited significantly higher one-year mortality (6; 20.7%) compared to those undergoing osteosynthesis (10; 3.8%) or arthroplasty (7; 5.7%) (p= 0.004) (Table 6). Increasing age (OR: 1.08; CI: 1.01-1.15; p= 0.034), COPD (OR: 5.24; CI: 1.38-19.90; p= 0.015), and cirrhosis (OR: 8.69; CI: 1.33-56.71; p=0.024) were identified as significant predictors of one-year all-cause mortality through logistic regression analysis (Table 8).

**Table 8:** Logistic regression predicting one-year all-cause mortality COPD: chronic obstructive pulmonary disease

Variable	OR (95% CI)	P-value
Age	1.08 (1.01-1.15)	0.034
Sex=female	0.75 (0.28-2.05)	0.580
Ethnicity=Hispanic	0.89 (0.25-3.16)	0.856
BMI	1.03 (0.94-1.12)	0.566
Displacement=displaced	1.23 (0.31-4.98)	0.769
Time to procedure (hours)	0.98 (0.93-1.04)	0.484
Procedure Type=arthroplasty	1.75 (0.59-5.18)	0.313
Procedure Type=conservative management	4.18 (0.76-22.90)	0.099
Hypertension	0.60 (0.18-1.99)	0.406
Prolonged immobilization	0.80 (0.31-2.06)	0.646
Diabetes mellitus	1.75 (0.64-4.73)	0.274
Functionally dependent health status	0.64 (0.21-1.98)	0.444
Dementia	1.37 (0.48-3.94)	0.554
Anticoagulant therapy	1.25 (0.44-3.55)	0.678
Osteoporosis	0.89 (0.31-2.60)	0.836
Current smoker	0.46 (0.05-4.20)	0.488
Congestive heart failure	0.24 (0.02-2.40)	0.226
COPD	5.24 (1.38-19.90)	0.015
Peripheral arterial disease	1.44 (0.28-7.44)	0.665
Chronic renal failure	0.93 (0.07-12.33)	0.954
Cirrhosis	8.69 (1.33-56.71)	0.024

Delayed surgery was associated with longer hospital stays. Patients surgically treated more than 48 hours after arrival had a mean stay of 7.96 days, compared to 6.44 days for those treated within 24 to 48 hours, and 5.73 days for those treated within 24 hours (p<0.001).

## Discussion

​​​​​​The RGV faces significant socioeconomic challenges, with over 25% of residents living below the poverty line compared to the US national average of 11.1%, and many experiencing limited access to healthcare due to high rates of uninsured and underinsured individuals [30,38]. Lower educational attainment contributes to disparities in health literacy and preventive care, with only 19% of adults aged 25 and older in the RGV having attained at least a bachelor’s degree, significantly lower than the national average of 37% [30,39]. Despite these challenges, the RGV is medically served by two Level 1 trauma centers, which play a critical role in managing high-acuity injuries, including hip fractures. Given these factors, this study provides an accurate representation of hip fractures in this medically underserved region.

The study population most commonly experienced intertrochanteric and femoral neck fractures, each resulting from distinct physiological and behavioral risk factors (Table 1) [22,32,40]. Fractures to the femoral neck have been linked to low calcaneal bone mineral density (BMD), poor functional status, and physical inactivity, while intertrochanteric fractures are associated with older age, poor health (age-related decline and comorbid conditions), and a history of osteoporosis [22,32,40]. A comparison analysis between this cohort and studies conducted by Löfman et al. and Asada et al. provided insight into regional and ethnic disparities in the occurrence of femoral neck (N) and intertrochanteric (IT) fracture patterns (Table 5) [36,37]. This predominantly Hispanic cohort showed a lower N/IT ratio, indicating a higher occurrence of intertrochanteric fractures among geriatric patients. Japan and Sweden were compared to explore the influence of ethnic, cultural, and environmental factors on fracture frequency and patterns, as their distinct genetic backgrounds, dietary habits, and lifestyles highlight disparities and protective factors unique to each group.

Femoral neck and intertrochanteric fractures require different surgical interventions based on their anatomy, blood supply, and biomechanics [13,14,17,20,41]. The surgical approach dictates post-operative management, including weight-bearing, rehabilitation, and recovery timelines [16,31,42,43]. Hip arthroplasty often enables earlier weight-bearing, while osteosynthesis may require prolonged protected ambulation and gradual physiotherapy, shaping recovery, and functional outcomes [31,42,43]. Activities of daily living are directly influenced by the fracture type sustained [4,20]. Preventing femoral neck fractures requires maintaining BMD and improving functional status, while addressing age-related health decline and strengthening bone health to reduce intertrochanteric fractures [40]. This necessitates targeted interventions, such as bone health education, balance training, home safety assessments, and the use of hip protectors [32,44].

A pre-fracture diagnosis of osteoporosis was present in 23.1% of patients, consistent with previous studies and highlighting the ongoing challenge of underdiagnosed osteoporosis (Table 2) [45-47]. Recent studies have identified a notably high prevalence of osteoporosis in the RGV, particularly within the Hispanic community [48-50]. Osteoporosis rates in the RGV exceeded the national average, with a prevalence of 12.3% among Hispanic ethnicity compared to 8.6% in Caucasians [48]. The lower percentage of pre-fracture osteoporosis diagnoses in geriatric males follows an important trend of men being underdiagnosed and undertreated for the condition [51-54]. These disparities emphasize the need for targeted screening and prevention strategies in this region and demographic group. The high prevalence of comorbidities such as diabetes, hypertension, and other chronic conditions in the RGV significantly contributes to the increased risk of hip fractures in the predominantly Hispanic geriatric population (Figure 1) [55-59]. These conditions adversely affect bone health, making bones more fragile and prone to fractures [6,8,9,15]. Additionally, the complications associated with diabetes and hypertension, such as poor circulation, neuropathy, and impaired balance, increase the likelihood of falls, further amplifying the risk of hip fractures among geriatric patients [3,6,8,9,15].

Fracture type is determined by the interaction between bone strength and impact forces. A shift in fracture type was observed with aging, as extracapsular fractures became increasingly common in both males and females (Table 3). Increased life expectancy, declining BMD, and falls collectively heighten the risk of hip fractures and influence their localization in geriatric patients [4,22,25]. As the geriatric population approaches the age of 80, the clinical focus should expand from solely managing bone health to prioritizing fall prevention.

Increased BMI heightens the risk of falls due to impaired balance and mobility, while greater body mass amplifies these forces [3]. A higher BMI was linked to a greater occurrence of intracapsular fractures, suggesting that body composition influences fracture type and localization through variations in biomechanical forces and soft tissue protection (Figure 2). Excess weight places mechanical stress on the femur, promoting growth in the diaphyseal and metaphyseal regions, including the calcar femorale, which supports the femoral neck and distributes loads [6,60,61]. The observation of more intracapsular fractures in this patient cohort with higher BMIs is noteworthy, as lower BMI has typically been associated with a greater risk of intracapsular fractures, and higher BMI was more commonly linked to extracapsular fractures [62-64]. Despite enhanced skeletal integrity and soft tissue cushioning, the femoral neck may remain vulnerable to concentrated stress, increasing the risk of intracapsular fractures.

Hispanic ethnicity was linked to a higher risk of extracapsular fractures (Table 4). Studies have shown that Hispanic populations can exhibit lower BMD, which predisposes them to fractures involving weaker bone structures, such as the extracapsular region of the proximal femur [4-6,44,48-50,65]. Vitamin D deficiency, compounded by genetic factors affecting vitamin D receptor function, lower supplementation rates, and darker skin pigmentation may further impair calcium absorption and bone strength, exacerbating fracture risk [65,66]. However, this aspect was not analyzed in the present study. Additionally, comorbidities like diabetes and obesity, which are prevalent in Hispanic populations, are linked to genetic and environmental factors that increase fall risk [67-69]. Sociocultural and economic influences, such as limited access to healthcare, dietary habits, and lower rates of osteoporosis screening, can delay the diagnosis and management of bone health issues [48,49,70]. Together, these genetic and sociocultural factors heighten the predisposition for extracapsular fractures in a predominantly Hispanic patient cohort, emphasizing the importance of culturally tailored prevention and management strategies [71].

An average time to surgery of 22.5 hours aligned with the hospital mandate to perform surgery within 24 hours while adhering to US national guidelines of a 48-hour window to avoid adverse outcomes (Table 6) [13,14,16,17,72,73]. While the time to surgery remained consistent across fracture types, delays in arrival-to-surgery time were associated with longer LOS. EMR chart reviews revealed that 15 patients experienced surgical delays exceeding 48 hours due to preoperative factors such as medical optimization and patient or family decision-making. Prolonged immobilization and diabetes mellitus emerged as significant risk factors for post-fracture complications (Table 7). Patients with prolonged immobilization prior to surgery had three times the odds of complications such as DVTs and pressure ulcers, while those with diabetes mellitus demonstrated an even higher risk. These findings underscore the importance of wound care management and glycemic control during the perioperative period [74]. Data showed patients with displaced fractures, anticoagulant therapy, and obesity had higher odds of complications but were not statistically significant.

The one-year all-cause mortality was relatively low compared to the 20%-30% mortality risk reported in the literature [5,7,10,14,16-20]. Age, chronic obstructive pulmonary disease (COPD), and cirrhosis emerged as significant predictors of mortality (Tables 6, 8). While prior studies examining hip fracture mortality across ethnic groups have shown higher mortality rates, these findings underscore important demographic disparities [75-80]. The lower mortality rate observed in this predominantly Hispanic cohort coincides with the Hispanic Paradox, a phenomenon in which Hispanic individuals achieve better health outcomes despite facing socioeconomic disadvantages [76,81-85]. This paradox has been attributed to protective factors such as strong social support networks and culturally ingrained health-promoting behaviors, which likely contributed to improved survival rates, even though 88.8% of study participants presented with multiple comorbidities or risk factors associated with their hip fractures [76,81-85]. Gaining insight into this phenomenon may be useful when extrapolating influential factors to improve outcomes and reduce national mortality rates across ethnicities. 

While this study provides valuable insights, several limitations must be acknowledged. The conservative management cohort consisted of patients who were not suitable surgical candidates due to unstable comorbidities or pre-existing functional limitations, both of which could independently impact outcomes. Additionally, patient LOS may have been prolonged due to lower insurance coverage rates within our cohort. Limited insurance and restricted access to rehabilitation services could have contributed to delays in securing appropriate post-hospital care, ultimately extending hospitalization. The retrospective design of this study also limits the ability to establish causality between risk factors and outcomes. Furthermore, although various comorbidities and lifestyle factors were considered, unmeasured confounders, such as socioeconomic status and medication use, may have influenced the results. Lastly, mortality outcome assessment was limited to a one-year follow-up period, and some patients had insufficient follow-up time to reach this milestone, potentially missing longer-term complications or mortality trends.

## Conclusions

The study emphasizes the distinct patterns and implications associated with hip fractures in a predominantly Hispanic geriatric patient cohort along the US-Mexico border. The comparison between extracapsular and intracapsular fractures underscores the complex interplay of ethnicity, chronic conditions, and socioeconomic factors contributing to these injuries. Recognizing the fracture type-specific risks and outcomes within this study population contributes to improving geriatric care across diverse communities. Implementing targeted prevention strategies, such as fall prevention programs, bone health education, and culturally tailored healthcare interventions, is crucial for reducing the incidence and impact of hip fractures in this vulnerable group.
